# On the definition of Landau constants in amplitude equations away from a critical point

**DOI:** 10.1098/rsos.180746

**Published:** 2018-11-14

**Authors:** Khanh Gia Pham, Sergey A. Suslov

**Affiliations:** Department of Mathematics, Swinburne University of Technology, John Street, Hawthorn, Victoria 3122, Australia

**Keywords:** amplitude expansion, Landau constants, low-dimensional projection

## Abstract

A weakly nonlinear stability analysis of shear flows based on amplitude expansion is re-examined. While it has been known that the condition required to define the coefficients of the resulting Stuart–Landau series representing the nonlinear temporal evolution of the most amplified Fourier component of a disturbance is not unique, we show that it can be formulated in a flexible generic form that incorporates different conditions used by various authors previously. The new formulation is interpreted from the point of view of low-dimensional projection of a full solution of a problem onto the space spanned by the basic flow vector and the eigenvector of the linearized problem. It is rigorously proven that the generalized condition formulated in this work reduces to a standard solvability condition at the critical point, where the basic flow first becomes unstable with respect to infinitesimal disturbances, and that it results in a well-posed problem for the determination of coefficients of Stuart–Landau series both at the critical point and a finite distance away from it. On a practical side, the generalized condition reported here enables one to choose the projection in such a way that the resulting low-dimensional approximate solution emphasizes specific physical features of interest via selecting the appropriate projection weight matrix without changing the overall asymptotic expansion procedure.

## 1. Introduction

Weakly nonlinear stability analysis of fluid flows is a mature area of research. It was introduced in its most well-known form in the 1960s in pioneering works [1,2] with a number of notable contributions throughout the 1970s, see [3–6] and references therein, to name a few. The essence of such an analysis is in the representation of the full solution of the governing equations as a sum of the so-called basic flow, which is a relatively simple solution of the governing equations obtained analytically or numerically, and perturbations. Linearization of a problem about the basic flow results in an eigenvalue problem for perturbations. Upon a suitable discretization a problem containing differential operators is typically converted to a generalized algebraic eigenvalue problem and the resulting eigenvectors determine the spatial distribution of perturbed flow quantities. However, the magnitude of such disturbances cannot be determined within the framework of a linearized problem so that the perturbation amplitude is taken as an unknown time-dependent factor multiplying the spatial eigenfunction. One of the main goals of a weakly nonlinear analysis is to model the temporal evolution of the disturbance amplitude *A*(*t*) and to determine whether and at what value it saturates. This leads to the derivation of the so-called amplitude equations of Stuart–Landau type [1,7]

1.1 $$\frac{\text{d}A}{\text{d}t}=\sigma A+A\sum_{k=1}^{\infty }K_{k}|A{|}^{2k}.$$

There have been suggested several ways for such a derivation with one of the most common methods using the multiple time scale expansion approach [8]. More recently, procedures based on a centre manifold reduction were also used and shown to produce identical results, however, at a much higher computational cost comparable with that of direct numerical computations, see [9]. This frequently makes the multiple scale expansion the method of choice when relatively inexpensive practical results in the finite neighbourhood of a critical point are of interest.

A crucial point in developing weakly nonlinear analysis is the choice of a small parameter that is used for producing asymptotic series approximating the full nonlinear solution of the problem. The relative parametric distance ‖*R* − *R*_c_‖/‖*R*_c_‖ ≪ 1, where *R* and *R*_c_ represent the sets of problems’ governing parameters at which the solution is required and of their critical values at which the basic flow first becomes unstable with respect to infinitesimal disturbances, respectively, is frequently chosen as the expansion parameter following the pioneering work [1]. This enforces a specific scaling of solution quantities near the critical point. In particular, the amplification rate of infinitesimal disturbances needs to be proportional to ‖*R* − *R*_c_‖/‖*R*_c_‖. The validity of such a rigid scaling is expected in the asymptotic vicinity of the critical point but cannot be guaranteed in practically important regimes characterized by small but finite values of ‖*R* − *R*_c_‖/‖*R*_c_‖. Moreover, such a parameter cannot be introduced in principle for flows that are always linearly stable such as pipe Poiseuille or plane Couette flows [10].

From a physical point of view, it appears more natural to take the magnitude of the perturbation amplitude itself as a small parameter. This leads to the so-called amplitude expansion [2,10]. It has been shown that its introduction does not rely on the proximity of the critical point or any rigid *a priori* scaling assumptions on solution quantities. In particular, in this approach the amplification rate of infinitesimal disturbances may happen to be, but does not have to be asymptotically small. The only validity condition for such a procedure is imposed by the requirement that the perturbation amplitude remains sufficiently small so that its powers form a set of elements with magnitudes progressively decreasing at a sufficiently fast rate to ensure the meaningful truncation of the asymptotic series (1.1) [11–13].

Despite this apparent advantage of using amplitude expansion there exists a very large body of literature, where parametric expansions about the critical point are favoured as the main tool of weakly nonlinear analysis. A comprehensive review of this field is outside the scope of the present paper, but we point an interested reader to studies reported in [8,14–16], where the comparison of parametric and amplitude expansion methods demonstrating the equivalence of various approaches near the critical point can be found and the methods are presented in a way similar to the amplitude expansion summarized in §2. One of the reasons the parametric expansion first introduced in [1] remains a popular choice is that algebraically it enables one to estimate the coefficients of the Stuart–Landau series (1.1) modelling a temporal evolution of the disturbance amplitude using the information from a critical point alone by employing the uniquely defined solvability condition (see equation (2.23) in §2.3). This, however, comes at a price of a reduced convergence range [17]. The parametric expansion approach about a critical point also suffers from a subtle inherent inefficiency. Before the method can be applied, the critical point in the problem parameter space has to be found. This is typically done iteratively by solving the linearized eigenvalue problem over a range of the parameter values *R* until *R*_c_ is found. Therefore, the eigenfunctions are readily available at the parametric point of interest *R* and can be used as the basis of the amplitude expansion. However, in order to cast the problem in the form suitable for the application of the solvability condition this readily available eigenfunction of interest is ignored and replaced by the one computed at the critical point.

By contrast, the amplitude expansion procedure summarized in §2 allows using the eigenfunction computed for the parametric values of interest away from a critical point directly. This, however, introduces the ambiguity demonstrated in §2.3 in supercritical regimes the definition of the eigenfunction amplitude becomes non-unique and requires an additional condition to be fixed. Such an ambiguity has been recognized from the inception of the method [2] and various authors proposed different ways of fixing it [9–11,13,18]. In the present paper, we show that previous individual attempts of fixing this ambiguity can be cast in a flexible general form that has a straightforward interpretation in the context of projecting fully nonlinear solutions onto the space spanned by the vector of the basic flow and the eigenvector of the linearized problem. The procedure enables one to effectively choose ‘the projection angle’ in such a way that the resulting low-dimensional solution is optimized to emphasize the desired aspects of the full solution. From a practical point of view, this, perhaps, is the main feature of the procedure described in this paper. We start with the idea first formulated in [19] and subsequently prove that the weakly nonlinear stability problem complemented with the specific projection criterion is well-posed at the critical point and away from it. Therefore, Landau constants in the Stuart–Landau series (1.1) can be efficiently evaluated taking into account the chosen meaning of the amplitude.

## 2. Summary of the expansion formalism and properties of the resulting operators

### 2.1. Basic flow

Consider a system of governing equations of Navier–Stokes type written in an appropriately non-dimensionalized form. Such a system of equations includes linear and nonlinear (at least quadratic) terms and represents the fundamental physical principles of conservation of momentum, mass and thermal energy and can include constitutive equations describing various fluid properties and processes taking place in it. The actual form of equations depends on the physical problem at hand and is not of importance for the analysis presented in this paper, but we will refer to the following set of non-dimensional equations describing a two-dimensional fluid flow between two infinitely wide and long differentially heated vertical plates, see [12] and §6 for details, as an illustration of the overall equation structure that is taken into account in the subsequent derivations:

2.1 $$\frac{\partial u}{\partial t}+u\cdot \nabla u=-\nabla p-\frac{Tg}{\text{Gr}}+\frac{1}{\text{Gr}}\nabla^{2}u,\;\nabla \cdot u=0$$

and

2.2 $$\frac{\partial T}{\partial t}+u\cdot \nabla T=\frac{1}{\text{GrPr}}\nabla^{2}T.$$

Here u=(u,v) is the vector of fluid velocity, *p* is the pressure, *T* is the fluid temperature, g=(0,−1) is the unit vector in the direction of gravity and *R* = (Gr, Pr) is the set of the governing non-dimensional parameters (Grashof and Prandtl numbers in this example). The fluid flow is considered in a domain that is bounded in at least one spatial (−1 ≤ *x* ≤ 1 in the above example) direction and unbounded in at least one other (−∞ < *y* < ∞ in the above example) direction. Assume that the above equations subjected to appropriate boundary conditions have a ‘simple’ steady solution $w_{00}={\left(u_{00},T_{00},p_{00}\right)}^{T}$ referred to as the basic flow. By ‘simple’, it is typically meant that for any fixed set of the governing parameter values *R* such a solution depends on the spatial coordinate in the direction of finite system extent (*x*), but not the one extending to infinity (translational symmetry in *y*). Symbolically, this is written as $w_{00}=w_{00}(x;R)$.

### 2.2. Linear stability and operators

Once the basic flow is found the question arises whether it is stable with respect to infinitesimal perturbations. In spatially extended systems, they can be conveniently given in the normal form $A(t)w_{11}(x)E+c.c.$, where *A*(*t*) is the time-dependent complex amplitude of disturbances with time evolution generally given by

2.3 $$\frac{\text{d}A}{\text{d}t}=F(A),$$

the term *E* ≡ exp(i*αy*) signifies the fact that the disturbance is 2**π**/*α* periodic in the extended direction, *α* is the spatial wavenumber and c.c. stands for complex conjugate. Substituting

2.4 $$w=w_{00}+Aw_{11}E+\text{c.c.}$$

into (2.1) and neglecting terms containing higher powers of amplitude one obtains a system of linear partial differential equations that can be written in a matrix operator form as

2.5 $$AA_{\alpha ;R}w_{11}E=\frac{\text{d}A}{\text{d}t}Bw_{11}E.$$

Here matrix operator $A_{\alpha ;R}$ represents terms involving spatial derivatives. This operator also contains governing parameters *R* appearing in the original equations. Matrix B arises from terms containing time derivatives so that for time-dependent problems $Bw_{11}\neq 0$. Typically, the governing equations can be written in such a form that this matrix does not contain the governing parameters, but it is singular as some of the governing equations, such as the continuity equation in system (2.1) does not contain time derivative explicitly. Thus B contains zero rows. This, however, does not affect the proofs and discussions presented in the subsequent sections as long as the condition $Bw_{11}\neq 0$ remains valid. In practice, equation (2.5) is solved numerically upon adopting a suitable discretization scheme for approximating spatial derivatives. As a result, matrix differential operators $A_{\alpha ;R}$ and B are converted to square *N* × *N* matrices with complex elements, where size *N* depends on the number of individual scalar functions forming solution w and the number of discretization points for each of them. Thus in what follows we will treat these operators as regular matrices and solution vectors as regular vectors.

Equation (2.5) can only be satisfied for any time if *F*(*A*) = σ*A* that is

2.6 $$\frac{\text{d}A}{\text{d}t}=\sigma A$$

and

2.7 $$A(t)=A_{0}\exp(\sigma t),$$

where σ is referred to as the complex amplification rate: σ = σ^*R*^ + *i*σ^*I*^. Equation (2.5) is then rewritten in the operator form as

2.8 $$(A_{\alpha ;R}-\sigma B)w_{11}\equiv L_{\alpha ,\sigma ;R}w_{11}=0.$$

The above has a non-trivial solution $w_{11}\neq 0$ only if σ is the eigenvalue of the operator $L_{\alpha ,\sigma ;R}$. When the problem domain is bounded (e.g. in *x* in the illustrative example considered above) the eigenvalue spectrum is discrete for each fixed value of wavenumber and governing parameters and the eigenvalues σ_*i*_(*α*; *R*), *i* = 1, 2, … can be arranged in the order of decreasing real part. The basic flow is deemed linearly unstable if the real part of at least the first of the so-sorted eigenvalues satisfies the condition $\sigma_{1\max}^{R}\equiv \max_{\alpha }(\sigma_{1}^{R})>0$, where the maximum is achieved at *α* = *α*_1max_. The value of the governing parameters *R* = *R*_c_ at which $\sigma_{1\max}^{R}=0$ is called critical. It corresponds to the bifurcation point at which the basic flow becomes linearly unstable. Of primary interest here is the 2*π*/*α*_1max_ periodic disturbance corresponding to the fastest growing small amplitude mode with σ = σ_1_ the temporal evolution of which we aim to investigate at the supercritical values of the governing parameter set *R*.

While the linearized consideration is capable of predicting the spatial shape of such a mode, its amplitude cannot be determined in the framework of linear analysis and this necessitates weakly nonlinear consideration. We outline it next for the situation when the basic flow becomes linearly unstable with respect to exactly one mode (and its complex conjugate), that is when $\sigma_{1}^{R}\geq 0>\sigma_{2}^{R}\geq \sigma_{3}^{R}\geq \ldots$. In doing so, we will rely on the following two properties of the linear operator $L_{\alpha ,\sigma ;R}$.

**Remark 2.1.**

The leading eigenvalue σ_1_ of the linear operator $L_{\alpha ,\sigma ;R}$ has multiplicity 1 so that the rank of the *N* × *N* operator $L_{\alpha ,\sigma ;R}$ is *N* − 1.

**Remark 2.2.**

For any complex number *s* such that $ℜ\{s\}=s^{R}>\sigma_{1\max}^{R}>0$ operator $L_{\alpha ,s}$ is non-singular.

### 2.3. Hierarchy of nonlinear terms and corresponding equations

Because of a quadratic nonlinearity of the governing equations, substitution of (2.4) in them produces terms that can be generally written as $|A{|}^{2}f_{20}$ and $A^{2}f_{22}E^{2}+\text{c.c.}$ They can only be balanced if (2.4) is extended to include functionally similar terms:

2.9 $$w=w_{00}+|A{|}^{2}w_{20}+(Aw_{11}E+A^{2}w_{22}E^{2}+\text{c.c.}).$$

It is easy to show that the additional terms must satisfy

2.10 $$|A{|}^{2}A_{0;R}w_{20}-\frac{\text{d}|A{|}^{2}}{\text{d}t}Bw_{20}=|A{|}^{2}f_{20}$$

and

2.11 $$A^{2}A_{2\alpha ;R}w_{20}-\frac{\text{d}A^{2}}{\text{d}t}Bw_{22}=A^{2}f_{22}.$$

Given (2.7) these become

2.12 $$L_{0,2\sigma^{R};R}w_{20}=f_{20}\;\text{and}\;L_{2\alpha ,2\sigma ;R}w_{22}=f_{22},$$

respectively. Since in (2.12) *α* = *α*_1max_ and σ = σ_1max_, we set $s^{R}=2\sigma_{1\max}^{R}>\sigma_{1\max}^{R}>0$ in remark 2.2 and conclude that the operators in the left-hand sides of the above equations are non-singular and remain so even when *R* → *R*_c_ and σ^*R*^ → 0. Therefore, unique solutions for $w_{20}$ and $w_{22}$ always exist.

Substituting (2.9) into the governing equations will produce yet another group of terms that are generally written as $A|A{|}^{2}f_{31}E+\text{c.c.}$ and $A^{3}f_{33}E^{3}+\text{c.c.}$ in the equations. Balancing them requires a further extension of (2.9):

2.13 $$w=w_{00}+|A{|}^{2}w_{20}+(Aw_{11}E+A^{2}w_{22}E^{2}+A|A{|}^{2}w_{31}E+A^{3}w_{33}E^{3}+\text{c.c.}).$$

In a way similar to that discussed above, we can show that a unique solution for $w_{33}$ is obtained from

2.14 $$A^{3}A_{3\alpha ;R}w_{33}-\frac{\text{d}A^{3}}{\text{d}t}Bw_{33}=A^{3}f_{33}$$

or

2.15 $$L_{3\alpha ,3\sigma ;R}w_{33}=f_{33},$$

since the operator in the left-hand side of (2.15) is non-singular in view of remark 2.2 and remains so when *R* → *R*_c_ and σ^*R*^ → 0. However, handling of equation for $w_{31}$ needs to be more delicate. It becomes

2.16 $$A|A{|}^{2}A_{\alpha ;R}w_{31}-\frac{\text{d}A|A{|}^{2}}{\text{d}t}Bw_{31}=A|A{|}^{2}f_{31}$$

or

2.17 $$L_{\alpha ,\sigma +2\sigma^{R};R}w_{31}=f_{31}.$$

If σ^*R*^ > 0, then in view of remark 2.2 the operator in the left-hand side of (2.17) is non-singular and the unique solution for $w_{31}$ can be found. However, if *R* → *R*_c_ and σ^*R*^ → 0, $L_{\alpha ,\sigma +2\sigma^{R};R}\to L_{\alpha ,\sigma ;R_{c}}$ and becomes singular so that the existence of solution $w_{31}$ cannot be guaranteed. Note that to this point no approximation has been introduced in the above procedure and all results obtained so far are exact. However, to resolve the potential unsolvability problem for $w_{31}$ the following approximation is required: it needs to be assumed that the evolution function *F*(*A*) can be represented in terms of an asymptotic series in amplitude

2.18 $$\frac{\text{d}A}{\text{d}t}=F(A)=\sigma A+KA|A{|}^{2}+\cdots ,$$

that can be meaningfully truncated after a finite number of terms (of particular interest here is truncation after a cubic term in amplitude that leads to the so-called cubic Landau equation [20], where *K* is known as Landau constant). This requires the perturbation amplitude to remain small yet this does not impose any other explicit restrictions. In particular, it does not necessitate any explicit scaling restrictions on the parametric distance from a critical point (σ^*R*^, *R*) = (0, *R*_c_) or, in fact, on the magnitude of σ^*R*^. Consequently, as stated in [10] such an approach termed amplitude expansion can in principle be applied for a weakly nonlinear analysis even of linearly stable flows such as plane Couette or circular pipe Poiseuille flows for which a critical point cannot be found.^1^

Adopting (2.18) instead of (2.6) does not affect the linearized equation (2.8) or unconditionally solvable hierarchical equations (2.12) and (2.15), but it leads to the appearance of an additional term in equation (2.17) that becomes

2.19 $$L_{\alpha ,\sigma +2\sigma^{R};R}w_{31}\equiv (L_{\alpha ,\sigma ;R}-2\sigma^{R}B)w_{31}=KBw_{11}+f_{31}.$$

Equation (2.19) requires different treatments in cases σ^*R*^ → 0 and σ^*R*^ ≠ 0.

In the limit of σ^*R*^ → 0, the existence of the solution $w_{31}$ can only be ensured by enforcing the so-called solvability condition. Consider the adjoint eigenvalue problem

2.20 $$L_{\alpha ,\sigma ;R}^{†}w_{11}^{†}\equiv (A_{\alpha ;R}^{H}-\sigma^{*}B^{H})w_{11}^{†}=L_{\alpha ,\sigma^{*};R}^{H}w_{11}^{†}=0,$$

where * denotes complex conjugation and the adjoint operator is defined with respect to some appropriately chosen inner product as

2.21 $$\langle w_{11}^{†},L_{\alpha ,\sigma ;R}w_{11}\rangle =\langle L_{\alpha ,\sigma ;R}^{†}w_{11}^{†},w_{11}\rangle =0.$$

Considering the inner product of the adjoint eigenfunction $w_{11}^{†}$ with (2.19) in the limit σ^*R*^ → 0, we obtain

2.22 $$0=K\langle w_{11}^{†},Bw_{11}\rangle +\langle w_{11}^{†},f_{31}\rangle$$

and, assuming that $\langle w_{11}^{†},Bw_{11}\rangle \neq 0$, the Landau constant is uniquely defined using the chosen inner product as

2.23 $$K=-\frac{\left\langle w_{11}^{†},f_{31}\right\rangle }{\left\langle w_{11}^{†},Bw_{11}\right\rangle }.$$

The derivation above demonstrates that the definition of the Landau constant is unique at a critical point (σ^*R*^, *R*) = (0, *R*_c_). However, if σ^*R*^ ≠ 0, the operator in the left-hand side of (2.19) is non-singular and the solution can be obtained for any value of *K*, which introduces the ambiguity at supercritical points with σ^*R*^ > 0 that are of main practical interest. Such an ambiguity was discussed in [10], where it was proposed to use an additional condition different to the above solvability condition to evaluate the Landau constant in supercritical regimes. However, the procedure was found to lead to numerical errors in the vicinity of the critical point because of the ill-conditioning of operator $L_{\alpha ,\sigma +2\sigma^{R};R}$ there. Subsequently, it was suggested in [19] to combine the tasks of finding $w_{31}$ and determining *K* into a single problem by adding an extra condition to the system. This was shown to work in practice, but no formal algorithmic proof was developed. In the next section, we present a generalization of the procedure for determining the Landau constant suggested in [19] along with a full proof of the algorithm that guarantees its uniform performance both at a critical point and a finite distance from it. We also prove that at a critical point the generalized procedure reduces to the application of a conventional solvability condition.

## 3. Evaluation of Landau constant

As follows from remark 2.2, system (2.19) is unconditionally solvable for any value of parameter *K* if σ^*R*^ > 0. To eliminate the ambiguity in defining the value of *K*, an additional condition is required. We choose to specify it in terms of the inner product

3.1 $$\langle w_{11},Mw_{31}\rangle \equiv w_{11}^{H}\cdot (Mw_{31})=(w_{11}^{H}M)\cdot w_{31}=0,$$

where the superscript ‘H’ denotes Hermitian (conjugate) transpose and bold symbols represent *N* component complex-valued column vectors. The *N* × *N* matrix operator M must satisfy the positivity condition

3.2 $$\langle w,Mw\rangle =w^{H}\cdot (Mw)=(w^{H}M)\cdot w>0,$$

for any complex valued vector w such that Mw≠0, but otherwise it is arbitrary.

Combining (2.19) and (3.1) leads to the extended system of dimension *N* + 1

3.3 $$\hat{L}{\hat{w}}_{31}={\hat{f}}_{31},$$

where

$$\hat{L}=\left[\begin{array}{cc} L_{\alpha ,\sigma ;R}-2\sigma^{R}B & -Bw_{11}\\ w_{11}^{H}M & 0 \end{array}\right],\;{\hat{w}}_{31}=\left[\begin{array}{c} w_{31}\\ K \end{array}\right],\;{\hat{f}}_{31}=\left[\begin{array}{c} f_{31}\\ 0 \end{array}\right],\;Mw_{11}\neq 0.$$

First, we show that matrix operator $\hat{L}$ is non-singular that is

3.4 $$\det\hat{L}\neq 0$$

for any value of σ^*R*^ so that the unique solution

$${\hat{w}}_{31}={\hat{L}}^{-1}{\hat{f}}_{31}$$

can be always found. We consider cases σ^*R*^ ≠ 0 and σ^*R*^ = 0 separately.

*Case* *1*: σ^*R*^ ≠ 0. We will prove (3.4) by contradiction.

**Proof.**

Suppose $\text{det}\;\hat{L}=0$. Then the columns of $\hat{L}$ are linearly dependent vectors and there exists the coefficient vector

3.5 $$\hat{d}=\left[\begin{array}{c} d_{1}\\ \vdots \\ d_{N+1} \end{array}\right]\neq \hat{0}$$

such that the linear combination of the column vectors

$$\hat{L}\hat{d}=0.$$

This leads to

3.6*a* $$(L_{\alpha ,\sigma ;R}-2\sigma^{R}B)d=d_{N+1}Bw_{11}$$

and

3.6*b* $$(w_{11}^{H}M)\cdot d=0,$$

where $d={\left(d_{1},\ldots ,d_{N}\right)}^{T}$. If *d*_*N*+1_ = 0, then it follows from (3.6a) that d=0 since the operator in the left-hand side is non-singular and thus $\hat{d}=\hat{0}$, which contradicts (3.5). Therefore, *d*_*N*+1_ ≠ 0 and without loss of generality we set *d*_*N*+1_ = 1. Then d is uniquely defined by

$$d=L_{\alpha ,\sigma +2\sigma^{R};R}^{-1}Bw_{11}.$$

Taking into account that by construction $L_{\alpha ,\sigma ;R}w_{11}=0$, it is easy to check that this unique solution of (3.6a) is

3.7 $$d=-\frac{1}{2\sigma^{R}}w_{11}.$$

By substituting (3.7) into (3.6b), we obtain $(1/2\sigma^{R})(w_{11}^{H}M)\cdot w_{11}=0$. Since $Mw_{11}\neq 0$, this contradicts (3.2). Thus, $\det\hat{L}\neq 0$ when σ^*R*^ ≠ 0. ▪

*Case* *2*: σ^*R*^ = 0. In this case,

3.8 $$L_{\alpha ,\sigma ;R}-2\sigma^{R}B=L_{\alpha ,\sigma ;R},$$

which is singular by construction. However, we will prove that

3.9 $$\det\hat{L}=-\langle w_{11}^{†},Bw_{11}\rangle \neq 0,$$

where $w_{11}^{†}$ is the eigenfunction of the adjoint problem defined as

3.10 $$L_{\alpha ,\sigma ;R}^{†}w_{11}^{†}\equiv (A_{\alpha ;R}^{H}-\sigma^{*}B^{H})w_{11}^{†}=L_{\alpha ,\sigma^{*};R}^{H}w_{11}^{†}=0.$$

**Proof.**

Consider the co-factor matrix
3.11
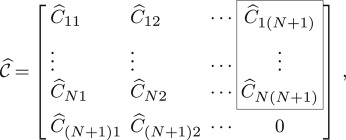
of the matrix operator $\hat{L}$, where ${\hat{C}}_{ij}={\left(-1\right)}^{i+j}\det({\hat{L}}_{ij})$ and ${\hat{L}}_{ij}$ is a *N* × *N* matrix formed by deleting the *i*th row and *j*th column from $\hat{L}$. The last element in (3.11) is zero because $\det L_{\alpha ,\sigma ;R}=0$ in *Case 2*. Introduce vector

$$c^{T}=({\hat{C}}_{1(N+1)},\ldots ,{\hat{C}}_{N(N+1)})$$

obtained from the last column in the matrix $\hat{C}$ (the framed part of (3.11)) by deleting the last element and compute $\det\hat{L}$ by expanding by the last column in $\hat{L}$

3.12 $$\det\hat{L}=-c^{T}\cdot (Bw_{11})=-\langle c^{*},Bw_{11}\rangle .$$

Further, we expand the individual co-factors ${\hat{C}}_{i(N+1)}$, *i* = 1, …, *N* by the last row in each matrix ${\hat{L}}_{i(N+1)}$ and obtain

$$c=C(M^{T}w_{11}^{*}),$$

where

3.13 $$C=\left[\begin{array}{cccc} C_{11} & C_{12} & \cdots & C_{1N}\\ \vdots & \vdots & \ddots & \vdots \\ C_{N1} & C_{N2} & \cdots & C_{NN} \end{array}\right]$$

is the co-factor matrix of matrix $L_{\alpha ,\sigma ;R}$, $C_{ij}={\left(-1\right)}^{i+j}\det(L_{ij})$ and $L_{ij}$ is a (*N* − 1) × (*N* − 1) matrix obtained by deleting the *i*th row and the *j*th column in $L_{\alpha ,\sigma ;R}$. This implies that

3.14 $$c^{T}=w_{11}^{H}MC^{T}.$$

Then upon right-multiplying by $L_{\alpha ,\sigma ;R}$ we obtain

3.15 $$c^{T}L_{\alpha ,\sigma ;R}=w_{11}^{H}MC^{T}L_{\alpha ,\sigma ;R}$$

**Remark 3.1**

(see section 0.8.2 in [22] for details). If $L_{\alpha ,\sigma ;R}$ is a square matrix with complex elements, then

3.16 $$C^{T}L_{\alpha ,\sigma ;R}=L_{\alpha ,\sigma ;R}C^{T}=(\det L_{\alpha ,\sigma ;R})I,$$

where I is the identity matrix of the same size as $L_{\alpha ,\sigma ;R}$.

Thus

3.17 $$c^{T}L_{\alpha ,\sigma ;R}=w_{11}^{H}M(\det L_{\alpha ,\sigma ;R})I=0^{T}$$

since $\det L_{\alpha ,\sigma ;R}=0$. Upon taking Hermitian transpose, (3.17) becomes

3.18 $${\left(c^{T}L_{\alpha ,\sigma ;R}\right)}^{H}=L_{\alpha ,\sigma^{*};R}^{H}c^{*}=L_{\alpha ,\sigma ;R}^{†}c^{*}=0.$$

We show next that c≠0 and thus

3.19 $$c^{*}=w_{11}^{†}.$$

**Proposition 3.2.**

c≠0.

**Proof.**

Suppose c=0 or ${\hat{C}}_{i(N+1)}={\left(-1\right)}^{i+j}\det({\hat{L}}_{i(N+1)})=0$, *i* = 1, …, *N*. Recollect that matrices ${\hat{L}}_{i(N+1)}$ consist of *N* − 1 rows of $L_{\alpha ,\sigma ;R}$

$$\{r_{1},r_{2},\ldots ,r_{i-1},r_{i+1},\ldots ,r_{N}\},$$

and $w_{11}^{H}M$. Since $\det({\hat{L}}_{i(N+1)})=0$, the rows of ${\hat{L}}_{ij}$ are linearly dependent and there exist a set of scalar coefficients

$$\left\{a_{i1},a_{i2},\ldots ,a_{i(i-1)},a_{i(i+1)},\ldots ,a_{iN},a_{i}\right\}$$

that are not zero simultaneously such that

$$a_{i1}r_{1}+a_{i2}r_{2}+\ldots +a_{i(i-1)}r_{i-1}+a_{i(i+1)}r_{i+1}+\cdots +a_{iN}r_{N}+a_{i}w_{11}^{H}M=0.$$

If *a*_*i*_ = 0 for all *i* = 1, …, *N*, then any selection of *N* − 1 rows from $L_{\alpha ,\sigma ;R}$ forms a linearly dependent set meaning that rank$(L_{\alpha ,\sigma ;R})<N-1$, which contradicts remark 2.1. Thus, the coefficient *a*_*i*_ ≠ 0 for some value of *i* in the range between 1 and *N*. Without loss of generality, it can be set to 1 to obtain

3.20 $$\begin{array}{rl} w_{11}^{H}M & =-a_{i1}r_{1}-a_{i2}r_{2}-\ldots -a_{i(i-1)}r_{i-1}-a_{i(i+1)}a_{i}r_{i+1}-\ldots -a_{iN}r_{N}\\ & =a^{T}L_{\alpha ,\sigma ;R}\;, \end{array}$$

where $a^{T}=-(a_{i1},a_{i2},\ldots ,a_{i(i-1)},0,a_{i(i+1)},\ldots ,a_{iN})$. Upon right-multiplying equation (3.20) by $w_{11}$, we obtain

$$w_{11}^{H}Mw_{11}=(a^{T}L_{\alpha ,\sigma ;R})w_{11}=a^{T}(L_{\alpha ,\sigma ;R}w_{11})=0.$$

This, however, contradicts condition (3.2). Thus, c≠0. ▪

The substitution of (3.19) into (3.12) then leads to

3.21 $$\det\hat{L}=-\langle c^{*},Bw_{11}\rangle =-\langle w_{11}^{†},Bw_{11}\rangle .$$

Now we prove that $\langle w_{11}^{†},Bw_{11}\rangle \neq 0$. To do so, we first demonstrate the validity of the following proposition.

**Proposition 3.3.**

Rank$(C^{T})=1$.

**Proof.**

Since rank$(L_{\alpha ,\sigma ;R})=N-1$ (see remark 2.1), this matrix contains *N* − 1 linearly independent column vectors. Then equation (3.16) becomes $C^{T}L_{\alpha ,\sigma ;R}=\det L_{\alpha ,\sigma ;R}I=O$, where O is a zero matrix of size *N* × *N*. This means that the dimension of the null space of $C^{T}$ is *n*≥*N* − 1, or rank$(C^{T})=N-n\leq 1$. Given that rank$(L_{\alpha ,\sigma ;R})=N-1$ there exists at least one non-zero minor of $L_{\alpha ,\sigma ;R}$ of size *N* − 1 so that the co-factor matrix $C^{T}$ of $L_{\alpha ,\sigma ;R}$ is non-zero and so is its rank. Thus, rank($C^{T})=1$. ▪

**Remark 3.4**

(**Full-rank factorization**, see section 0.4.6 in [22] for details). Let $C^{T}$ be an *N* × *N* matrix with complex elements. If rank$(C^{T})=1$ then there exist two non-zero column vectors u and v such that $C^{T}=uv^{T}$.

From equation (3.16), we obtain

3.22 $$L_{\alpha ,\sigma ;R}C^{T}=L_{\alpha ,\sigma ;R}uv^{T}=(L_{\alpha ,\sigma ;R}u)v^{T}=O.$$

Since $v^{T}\neq 0$, $L_{\alpha ,\sigma ;R}u=0$. Moreover, since u≠0 there exists a non-zero constant *γ*_1_ such that $u=\gamma_{1}w_{11}$.

Similarly, from equation (3.16) we obtain

3.23 $$C^{T}L_{\alpha ,\sigma ;R}=uv^{T}L_{\alpha ,\sigma ;R}=u{\left(L_{\alpha ,\sigma ;R}^{†}v^{*}\right)}^{T}=O.$$

Since u≠0, $L_{\alpha ,\sigma ;R}^{†}v^{*}=0$ and since v≠0 there must exist a non-zero constant *γ*_2_ such that $v^{*}=\gamma_{2}w_{11}^{†}$. Therefore,

$$C^{T}=\gamma w_{11}w_{11}^{†H},\;\gamma =\gamma_{1}\gamma_{2}\neq 0.$$

Differentiating the determinant of $L_{\alpha ,\sigma ;R}$ with respect to σ (see section 0.8.10.11 in [22]) we obtain

$$\frac{\text{d}}{\text{d}\sigma }\det L_{\alpha ,\sigma ;R}=\text{tr}\left(C^{T}\frac{\text{d}}{\text{d}\sigma }L_{\alpha ,\sigma ;R}\right),$$

where tr(⋅) denotes trace of the matrix expression in parentheses. Since $L_{\alpha ,\sigma ;R}=A_{\alpha ;R}-\sigma B$, $(\text{d}/\text{d}\sigma )L_{\alpha ,\sigma ;R}=-B$. This leads to

3.24 $$\frac{\text{d}}{\text{d}\sigma }\det L_{\alpha ,\sigma ;R}=-\text{tr}(C^{T}B)=-\gamma \;\text{tr}(w_{11}w_{11}^{†H}B).$$

Since the trace of a matrix product does not depend on the order of multiplication,

$$\text{tr}(w_{11}w_{11}^{†H}B)=\text{tr}(Bw_{11}w_{11}^{†H}).$$

Let $w_{11}={\left(m_{1},\ldots ,m_{N}\right)}^{T}$, $w_{11}^{†}={\left(m_{1}^{†},\ldots ,m_{N}^{†}\right)}^{T}$ and denote the elements of matrix B by *b*_*ij*_, *i*, *j* = 1, 2, …, *N*. Then

$${\left(Bw_{11}\right)}^{T}={\left(\sum_{\;j=1}^{N}b_{1j}m_{j},\sum_{\;j=1}^{N}b_{2j}m_{j},\ldots ,\sum_{\;j=1}^{N}b_{Nj}m_{j}\right)}^{T}$$

and

$$(Bw_{11})w_{11}^{†H}=\left[\begin{array}{cccc} m_{1}^{†*}\sum_{\;j=1}^{N}b_{1j}m_{j} & \;m_{2}^{†*}\sum_{\;j=1}^{N}b_{1j}m_{j} & \;\cdots & \;m_{N}^{†*}\sum_{\;j=1}^{N}b_{1j}m_{j}\\ \vdots & \vdots & \;\cdots & \vdots \\ m_{1}^{†*}\sum_{\;j=1}^{N}b_{kj}m_{j} & \;m_{2}^{†*}\sum_{\;j=1}^{N}b_{kj}m_{j} & \;\cdots & \;m_{N}^{†*}\sum_{\;j=1}^{N}b_{kj}m_{j}\\ \vdots & \vdots & \;\cdots & \vdots \\ m_{1}^{†*}\sum_{\;j=1}^{N}b_{Nj}m_{j} & \;m_{2}^{†*}\sum_{\;j=1}^{N}b_{Nj}m_{j} & \;\cdots & \;m_{N}^{†*}\sum_{\;j=1}^{N}b_{Nj}m_{j} \end{array}\right]$$

and then equation (3.24) assumes the form

3.25 $$\frac{\text{d}}{\text{d}\sigma }\det L_{\alpha ,\sigma ;R}=-\gamma \sum_{\;j=1}^{N}m_{j}^{†*}\sum_{k=1}^{N}b_{\;jk}m_{k}=-\gamma \langle w_{11}^{†},Bw_{11}\rangle .$$

As stated in remark 2.1 the algebraic multiplicity of eigenvalue σ is equal to 1. Thus the characteristic polynomial $\;p(t)=\det L_{\alpha ,t}$ is factored as

$$\;p(t)=(t-\sigma ){\;p}_{1}(t),$$

where *p*_1_(σ) ≠ 0. Then it follows that:

$$\frac{\text{d}}{\text{d}\sigma }\det L_{\alpha ,\sigma ;R}={\left.\frac{\text{d}\;p}{\text{d}t}\right|}_{t=\sigma }=-{\;p}_{1}(\sigma )\neq 0.$$

Hence $\langle w_{11}^{†},Bw_{11}\rangle \neq 0$ and we finally conclude that $\det\hat{L}\neq 0$ when σ^*R*^ = 0. ▪

The main result of the above derivation is that a unique solution of (3.3) can always be found because the matrix operator in its left-hand side is non-singular. In particular, a unique (for a chosen weight matrix M) value of Landau coefficient can be obtained using Cramer’s rule

3.26 $$K=\frac{\det\hat{S}}{\det\hat{L}},$$

where $\hat{S}$ is obtained from $\hat{L}$ by replacing $-Bw_{11}$ with $f_{31}$ for both cases σ^*R*^ ≠ 0 and σ^*R*^ = 0.

**Corollary 3.5.**

*In the limiting case of* σ^*R*^ = 0 (*critical or bifurcation point), the Landau coefficient is independent of the choice of the weight matrix* M *and is defined via the conventional solvability condition*.

**Proof.**

If σ^*R*^ = 0, the numerator in (3.26) becomes

$$\hat{S}=\left[\begin{array}{cc} L_{\alpha ,\sigma ;R} & f_{31}\\ w_{11}^{H}M & 0 \end{array}\right].$$

Since the only difference between $\hat{S}$ and $\hat{L}$ is due to the replacement of the element $-Bw_{11}$ with $f_{31}$, the procedure identical to that used to derive (3.21) leads to $\det\hat{S}=\langle w_{11}^{†},f_{31}\rangle$, which can be formally obtained by replacing $-Bw_{11}$ with $f_{31}$ in (3.21). Therefore, (3.26) becomes (2.23). Note that the adjoint eigenfunction $w_{11}^{†}$ is defined up to an arbitrary multiplicative constant. Since we have shown that $\langle w_{11}^{†},Bw_{11}\rangle \neq 0$ it can be chosen in such a way that $-\langle w_{11}^{†},Bw_{11}\rangle =1$. Then

$$K=\langle w_{11}^{†},f_{31}\rangle ,$$

which is independent of M and is identical to the definition of Landau coefficient using a conventional solvability condition (2.23). ▪

## 4. Interpretation of the procedure

**Figure 1.**
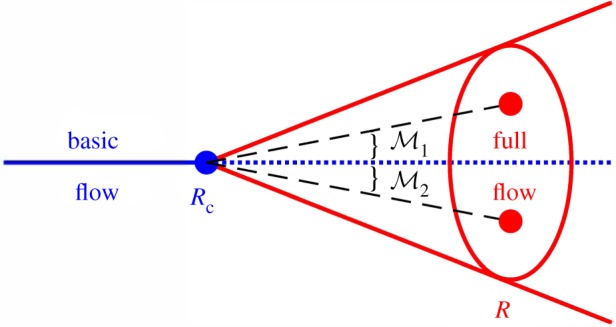
Schematic diagram of low-dimensional projection of supercritical flow solutions enforced by the procedure of §§2 and 3.

To interpret the main results of §3 refer to a schematic diagram shown in figure 1. Parametric point *R*_c_ (critical or bifurcation point) separates two qualitatively different regions. In the case of supercritical bifurcation for *R* < *R*_c_ a ‘simple’ basic flow solution to the problem at hand exists, while for *R* > *R*_c_ the full flow solution containing basic flow and its perturbations is found. The two types of solution have different dimensionalities: basic flow is shown by a segment of the horizontal line to the left of *R*_c_ while the full solution is represented by the conical region to the right of *R*_c_. To the leading order, the amplitude expansion procedure described in §2 projects the full flow solution onto a space spanned by the vector of the basic flow $w_{00}$ and the eigenvector $w_{11}$ of a linearized problem (2.8). The two principal points need to be emphasized here. First, no assumption of the closeness to the critical point is made, that is the condition ‖*R* − *R*_c_‖/‖*R*_c_‖ ≪ 1 commonly used in weakly nonlinear stability studies is not enforced here. Second, the eigenfunction used in the outlined projection procedure is evaluated at the parametric point of interest *R* rather than at the critical point *R*_c_. It corresponds to the fastest growing perturbation at point *R* rather than to the neutral disturbance at *R*_c_ frequently chosen for algebraic convenience in stability studies. As argued in [17], this choice is physically more relevant and it improves the convergence properties of the resulting asymptotic series.

The full solution of a problem at the parametric point *R* of interest is shown by an elliptic region in figure 1. The procedure outlined in the previous sections essentially selects a low-dimensional projection of this solution (circles inside the ellipse) by specifying the ‘angle’ of projection schematically represented by the slope of the dashed lines at the intersection of which with the ellipse the projected solutions lie. Such an ‘angle’ is defined by the properties of the used inner product 〈 · · · , · · ·〉 and, more specifically, by the structure of matrix M. Different projections corresponding to different matrices $M_{1}$ and $M_{2}$ (see the dashed lines with different slopes in figure 1) ‘puncture’ distinct points in the ellipse meaning that the outlined projection procedure can lead to different results away from the critical point. Yet when approaching the critical point in the limit *R* → *R*_c_ all projections must result in the same solution obtained using the solvability condition. In the view of the above, when applying the reduction procedure a finite distance away from the critical point one needs not only to perform a formal amplitude expansion, but also specify the ‘angle’ under which the desired result should be viewed. To clarify this point, we consider several examples of projections reported in the literature applied to three- or five-component solution $w_{31}^{T}=(u_{31},v_{31},p_{31})$ or $w_{31}^{T}=(u_{31},v_{31},w_{31},\theta_{31},p_{31})$, where *u*_31_, *v*_31_, *w*_31_, *θ*_31_ and *p*_31_ contain discretized values of the flow velocity components, temperature and the pressure.

(i) All elements of M are zero apart from *M*_*ii*_ = 1 for some *i* so that $Mw_{31}={\left(0,0,\ldots ,0,{\left(u_{31}\right)}_{i},0,\ldots ,0\right)}^{T}$. This condition was used in the work of Herbert [10]. In this case, the higher order in amplitude terms are chosen in such a way that the perturbation velocity component *u*_31_ at point *x*_*i*_ is set to zero so that in the vicinity of this particular point the perturbation of this specific velocity component is given completely by *Au*_11_(*x*_*i*_). As was shown in [17], the results of such a projection depend on a (subjective) choice of the ‘pinning’ point *x*_*i*_. This inherently local procedure would also break if *u*_11_(*x*_*i*_) and/or *u*_31_(*x*_*i*_) happen to be zero so that the compulsory condition (3.2) is violated.
(ii) In our earlier studies [18,23], M=I was chosen that corresponds to the global orthogonality condition $\langle w_{11},w_{31}\rangle =0$. As was demonstrated in [17] such a choice removes the ambiguity associated with a subjectivity of choosing a ‘pinning’ point mentioned above. It also improves the convergence properties of the resulting asymptotic series. To see that rearrange (2.13) to read
  4.1 $$w=w_{00}+A(w_{11}+|A{|}^{2}w_{31}+\cdots )E+\cdots .$$
  Asymptotically, the term in parentheses represents the fundamental disturbance harmonic given by an eigenfunction $w_{11}$ of the linearized problem and its higher-order distortion $w_{31}$ due to nonlinearity, with the leading term $Aw_{11}E$ being of primary interest. If $\langle w_{11},w_{31}\rangle =0$, the leading-order approximation $w=w_{00}+Aw_{11}E$ is not modified by the addition of higher order in amplitude terms as they are guaranteed to be orthogonal vectors. Essentially, (4.1) becomes an orthogonal expansion and its convergence properties improve.
(iii) In [21], M was chosen to be the identity matrix $I_{u,v}$, where the diagonal elements corresponding to pressure *p* were set to zero so that $\langle w,Mw\rangle =u^{2}+v^{2}$, which has the meaning of kinetic energy. Such a choice preserves the benefits of the orthogonal expansion but produces the expansion that is optimal for capturing the kinetic energy of perturbations. This is useful when weakly nonlinear stability analysis results are compared with experimental flow data where velocity fields are measured directly while the pressure fields are not recorded (e.g. [24]).
(iv) When integral spectral collocation methods are used as, for example, in [18,23], where the vector of unknown quantities is written in terms of a vector of their highest derivatives present in the governing equations multiplied by a spectral integration matrix (e.g. [25]), the diagonal identity matrices in the above examples become block-diagonal and so does matrix M.
(v) The possibility of using a less conventional normalization condition for the disturbance amplitude was mentioned in [26] in the context of Rayleigh–Bénard problem: 〈*v*_11_, *θ*_31_〉 = 0, where the solution vector $w={\left(u,v,w,\theta ,p\right)}^{T}$ consists of the velocity components *u*, *v* and *w*, temperature *θ* and pressure *p*. While the physical meaning of such a normalization has not been discussed, formally it can be cast in the form of (3.1) with
  $$M=\left[\begin{array}{ccccc} 0 & 0 & 0 & 0 & 0\\ 0 & 0 & 0 & 1 & 0\\ 0 & 0 & 0 & 0 & 0\\ 0 & 0 & 0 & 0 & 0\\ 0 & 0 & 0 & 0 & 0 \end{array}\right].$$

## 5. An alternative condition

So far, a procedure for calculating Landau constants using condition (3.1) was discussed, in which the orthogonality of the higher-order perturbations with the eigenfunction $w_{11}$ or its selected components was used and interpreted from a physical point of view. The main result was that such a procedure automatically reduces to the use of a standard solvability condition involving the adjoint eigenfunction at a critical point. For completeness of the discussion, we should mention here that it is possible to formally apply this condition away from a bifurcation point enforcing

5.1 $$\langle w_{11}^{†},Bw_{31}\rangle \equiv w_{11}^{†H}\cdot (Bw_{31})=(w_{11}^{†H}B)\cdot w_{31}=0.$$

as has been done in [12]. In this case, M=B and (3.3) is transformed to a different form

5.2 $$\hat{L}=\left[\begin{array}{cc} L_{\alpha ,\sigma ;R}-2\sigma^{R}B & -Bw_{11}\\ w_{11}^{†H}B & 0 \end{array}\right].$$

The proof that $\hat{L}$ in expression (5.2) is non-singular is similar to that presented in §3 and is not repeated here. The meaning of the corresponding amplitude expansion in this case is that the full flow solution is projected onto a space spanned by $w_{00}$ and $w_{11}^{†}$. While such a projection is formally possible, its physical interpretation away from the critical point is not clear. In this context, the formulation considered in §3 that enables one to choose upfront a type of projection suited for particular practical purposes such as those listed in §4 is more flexible and thus may be preferred.

## 6. Illustrative example

**Figure 2.**
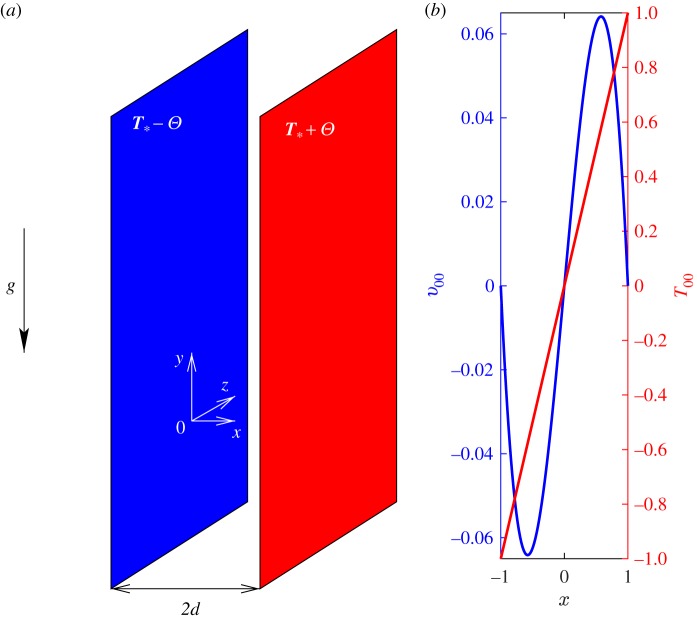
Sketch of a physical set-up of (*a*) and basic flow temperature and velocity profiles (*b*) for an illustrative example of convection between two differentially heated walls.

In this section, we illustrate the numerical results obtained for various choices of M in the well-studied example of natural convection flow of fluid with Prandtl number Pr = 7.5 contained between two vertical plates placed at non-dimensional positions *x* = ±1 and maintained at uniform non-dimensional temperatures *T*(±1) = ±1. The sketch of a physical set-up and non-dimensional basic flow solutions are shown in figure 2. The solution vector in this case consists of four elements $w^{T}=(u,v,T,p)$. Complemented with no-slip/no-penetration boundary conditions for velocities the system of equations (2.1) and (3.1) admits a steady basic flow solution of the form

6.1 $$u_{00}(x)=0,\;v_{00}(x)=\frac{1}{6}x(1-x^{2}),\;T_{00}(x)=x,\;p_{00}=\text{const.}$$

Such a simple flow becomes linearly unstable with respect to stationary *y*-periodic disturbances forming rolls with the axes in the mid-plane of the layer and wavenumber *α*_c_ = 1.383 at Gr_c_ = 491.8. Note that these values differ somewhat from *α*_c_ = 1.414 and Gr_c_ = 492.3 reported in [12] and are presumed to be more accurate given that they were obtained here using a spectral [25,27] rather than finite difference approximation.

**Figure 3.**
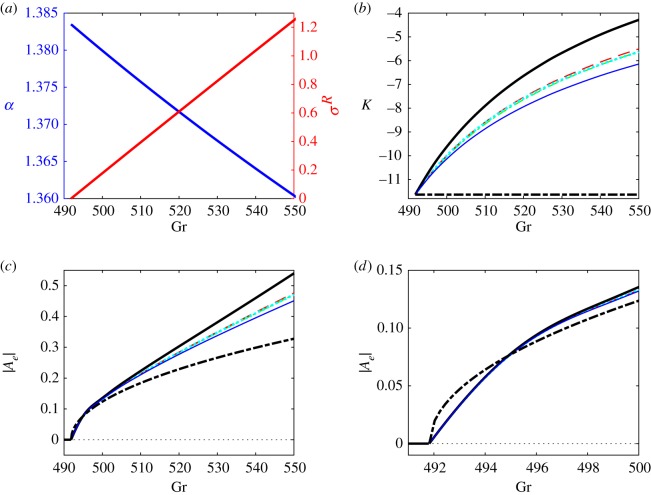
Stability characteristics and equilibrium amplitudes for a supercritical convection flow between differentially heated vertical plates. (*a*) Wavenumber *α* and linear amplification rate σ^*R*^, (*b*) Landau constants, (*c*) and (*d*) amplitudes of the most amplified perturbations as functions of Grashof number Gr. Results computed for Cases 1–3 discussed in §4 are shown by the black thick solid, green thin dash-dotted and red dashed lines, respectively. The projection results taking into account only fluid temperature are shown by the cyan thick dotted lines and the results of projection discussed in §5 are shown by the blue thin solid lines. The thick black dash-dotted lines correspond to evaluations using Landau constant determined at the critical point.

Because the developing instability is stationary, the linear amplification rate remains real (σ = σ^*R*^) and so does Landau constant *K* (figure 3*a*,*b*). In supercritical regimes, the wavenumber corresponding to the largest value of σ^*R*^ decreases and σ^*R*^ increases mostly linearly with Grashof number. The numerical values of Landau constant *K* are computed for the eigenfunctions of the linearized problem normalized to satisfy *u*_11_(0) = 1. They remain negative indicating the supercritical nature of bifurcation. However, the *K* values deviate significantly (and nonlinearly) from the value determined at the critical point in supercritical regimes. The comparison of the values of Landau constant *K* and equilibrium perturbation amplitude $|A_{e}|=\sqrt{-\sigma^{R}/K}$ obtained using various weight matrices M and approaches is given in figure 3*b*–*d*. It enables one to make the following observations.

Consistent with the proof given in §3 the use of all weight matrices M leads to the same value of Landau constant at the bifurcation point. This value *K* = −11.632 is identical to that obtained from a solvability condition (2.23) involving the adjoint eigenfunction. This value is also close to the one obtained using the correlation formulae $K=-720({\text{Pr}}^{1.325}/{\text{Gr}}_{c}\alpha_{c}^{2})\approx -11.050$ suggested in [15]. The values of *K* = −10.102 and *K* = −6.153 that we calculated following the procedure outlined in §5 (see the thin solid lines in figure 3) by fixing *α*_0_ = 1.414 for supercritical values of Gr = 500 and Gr = 550 as was done in [12] agree with their values of *K* = −10.100 and *K* = −6.151 (obtained upon rescaling the values given in Table I in [12] as $K\to K/(\text{Gr}\alpha_{0}^{2})$, which results from a different normalization of the eigenfunctions of a linearized problem used here).

As expected, in the close vicinity of the critical points all projections based on various weighted inner products produce numerical estimations of the perturbation amplitude that are virtually indistinguishable from each other (figure 3*d*). However, they all differ noticeably from the amplitude predictions based on Landau constant evaluated at a critical point shown by the thick dashed-dotted line. This is due to an inherent inconsistency of the way such a prediction is obtained. While its core assumption is that *K* remains constant for supercritical values of Gr, see the thick dashed-dotted line in figure 3*b*, to estimate non-trivial values of the amplitude $|A_{e}|=\sqrt{-\sigma^{R}/K}$ one has to use the actual supercritical values of σ^*R*^ computed for Gr > Gr_c_ as shown in figure 3*a*.

Further away from a critical point the amplitude estimations based on different weighted products produce numerically different results. Again, this is expected because the ‘effective dimensionality’ of the full problem solution increases, see the oval region in figure 1, while by its very nature each low-dimensional projection can emphasize only a limited subset of full flow features. Therefore, it becomes important to specify which solution characteristics are of the main interest in a particular physical context and embed this focus in the projection procedure from the outset. For example, in studies of isothermal fluid flows the dynamic characteristics of a perturbed flow are of primary interest [24] so that the projection selecting the kinetic energy [21] could be preferred, see Case 3 in §4. On the other hand, the main practical interest in convection problems such as the one considered in this section is the heat flux across the fluid layer. It is characterized by the average Nusselt number that is the ratio of the total heat flux to its conduction component. In terms of weakly nonlinear theory presented here, it is given by

6.2 $$\text{Nu}=1+|A_{e}{|}^{2}\frac{\text{d}T_{20}}{\text{d}x},$$

where the temperature derivative is evaluated at one of the walls at *x* = ±1. Therefore, the use of the weight matrix

**Figure 4.**
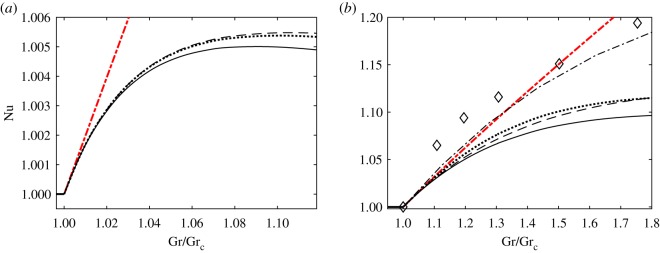
Nusselt number estimation using equation (6.2) and various weight matrices for (*a*) Pr=7.5 and (*b*) Pr = 0.71. The results obtained for M given by (6.3) are shown by the dotted lines. The solid lines depict results obtained following the procedure outlined in §5 (reproduced with permission from [12] for Pr = 7.5 in (*a*)). The results of projection emphasizing kinetic energy (Case 3 in §4) are shown by the dashed lines. The thick red dashed-dotted lines depict the estimation based on the data computed at the critical point while the thin dashed-dotted line in (*b*) shows experimental results of [28]. The diamonds correspond to direct numerical simulation of [29].

6.3 $$M=\left[\begin{array}{cccc} 0 & 0 & 0 & 0\\ 0 & 0 & 0 & 0\\ 0 & 0 & 1 & 0\\ 0 & 0 & 0 & 0 \end{array}\right],$$

that selects the perturbation temperature and downplays the other components of the solution may be preferred. The behaviour of the so-computed Nusselt number in supercritical regimes is demonstrated in figure 4*a* by the dotted line. It is virtually indistinguishable from that obtained by following the procedure outlined in §5 and previously used in [12] near the critical point, but the values obtained using these two approaches deviate somewhat further away from it. The dashed line depicts results obtained using a weight matrix emphasizing kinetic energy of the perturbation. They are close to but not identical to predictions based on (6.3).

To compare the Nusselt number estimations with DNS and experimental data available in the literature, we also computed the results for Pr = 0.71. They are shown in figure 4*b*. As expected, the low-dimensional projection results underestimate those of direct numerical simulations [29] and experiments [28] because they only take into account one fastest growing Fourier component with a fixed wavenumber while Fourier components covering a finite wavenumber range are present in supercritical regimes (see also discussion in [18]). Nevertheless, the agreement is reasonable^2^ and the closest match with experimental data is indeed obtained by choosing the weight matrix (6.3) emphasizing the temperature distribution in the disturbed flow. Qualitatively, after the quick initial growth near the bifurcation point the value of Nusselt number levels out. Such a behaviour is captured in the discussed projection by taking into account the variation of spatial eigenfunctions in supercritical regimes. At the same time, the estimation based on the data computed at the critical point alone is only capable of predicting the slope of the Nusselt number curve at the bifurcation point, see the thick red dashed-dotted lines in figure 4*a*, but the corresponding linear Nusselt number behaviour fails to follow the experimentally observed trends of levelling the Nusselt number curves away from a bifurcation point.

## 7. Conclusion

In this study, we revisited the procedure of amplitude expansion in the context of weakly nonlinear stability theory of flows arising in extended domains with at least one finite dimension. We emphasized the application of this procedure at parametric points located finite distance away from the critical point where the real amplification rate of infinitesimal disturbances becomes zero. We confirmed that the definition of the perturbation amplitude in this case is not unique and its meaning has to be specified from the outset to obtain meaningful physical interpretation of results. We demonstrated that this can be done by introducing an appropriate orthogonality condition with respect to a weighted inner product. The non-uniqueness of the amplitude definition offers an opportunity for choosing it in such a way that the resulting low-dimensional projection of the full solution emphasizes its specific features (e.g. kinetic energy or heat flux) that are of interest in a particular physical context. The current procedure is a generalization of those previously suggested in [10,12,18] and contains them as special cases. The main outcome of the current work is that the computational procedure for evaluating Landau constants in supercritical regimes finite distance away from a critical point, the idea of which was initially formulated in [19], has been put on a firm ground by formal proof of the facts that it does not result in any singularities and automatically recovers standard solvability condition at a critical point. While the work has focused on the evaluation of the first Landau constant appearing at the third order of amplitude, exactly the same procedure can be uniformly applied to evaluate Landau constants at higher orders of disturbance amplitude. Finally, we note that the procedure for evaluating Landau constants discussed here is also applicable for amplitude expansions in subcritical regimes (e.g. when basic flow is subject to a subcritical bifurcation at a critical point or when the basic flow remains stable with respect to infinitesimal disturbances). However, in this case special care needs to be taken in treating subcritical resonances that occur between decaying instability modes, see [3,11,18]. It follows from the presented proof that such resonances cannot arise in supercritical systems, but one has to be mindful of them in subcritical regimes because $L_{\alpha ,\sigma +2\sigma^{R};R}$ can in principle become singular if σ^*R*^ < 0. If this occurs, a system of coupled amplitude equations accounting for the resonant mode interaction needs to be considered, as discussed in [21]. A systematic procedure of resolving second and higher-order subcritical resonances suggested there can be used to derive a system of coupled Landau equations modelling the evolution of resonating subcritical modes. The computational procedure formulated here does not require any modifications to be used for evaluating Landau coefficients of such a system.

## Footnotes

1 In this case, σ^*R*^ < 0 so that remark 2.2 does not necessarily hold and the issue of subcritical resonances may arise, see, for example, [3,21] and references therein for a more detailed discussion.

2 Note that both DNS and experimental results are obtained for fluid layers of large but finite height and that the value of Nusselt number decreases with the height of the cavity. Therefore, it is expected that in the limit of an infinite vertical layer considered here both numerical and experimental results would extrapolate to lower values so that the difference between the data in figure 4*b* would be smaller.

## Data accessibility

This article has no additional data.
